# Biodistribution, Uptake and Effects Caused by Cancer-Derived Extracellular Vesicles

**DOI:** 10.5772/60522

**Published:** 2015-03-25

**Authors:** Lilite Sadovska, Cristina Bajo Santos, Zane Kalniņa, Aija Linē

**Affiliations:** 1 Latvian Biomedical Research and Study Centre, Riga, Latvia; 2 Faculty of Biology, University of Latvia, Riga, Latvia

**Keywords:** Extracellular vesicles, biodistribution, trafficking, tumour microenvironment, immunosuppression, metastatic niche

## Abstract

Extracellular vesicles (EVs) have recently emerged as important mediators of intercellular communication. They are released in the extracellular space by a variety of normal and cancerous cell types and have been found in all human body fluids. Cancer-derived EVs have been shown to carry lipids, proteins, mRNAs, non-coding and structural RNAs and even extra-chromosomal DNA, which can be taken up by recipient cells and trigger diverse physiological and pathological responses. An increasing body of evidence suggests that cancer-derived EVs mediate paracrine signalling between cancer cells. This leads to the increased invasiveness, proliferation rate and chemoresistance, as well as the acquisition of the cancer stem cell phenotype. This stimulates angiogenesis and the reprogramming of normal stromal cells into cancer-promoting cell types. Furthermore, cancer-derived EVs contribute to the formation of the pre-metastatic niche and modulation of anti-tumour immune response. However, as most of these data are obtained by *in vitro* studies, it is not entirely clear which of these effects are recapitulated *in vivo*. In the current review, we summarize studies that assess the tissue distribution, trafficking, clearance and uptake of cancer-derived EVs *in vivo* and discuss the impact they have, both locally and systemically.

## 1. Introduction

Extracellular vesicles (EVs) are a heterogeneous population of nanosized membrane vesicles that are released in the extracellular space by almost all normal and cancer cell types. Currently, three broad categories of EVs have been defined. These are based on the mode of biogenesis: (i) exosomes, (ii) microvesicles and (iii) apoptotic bodies [1]. Exosomes are EVs of endocytic origin, which range from 50-150 nm in diameter. These are released into the extracellular environment by a fusion of the multivesicular bodies (MVBs) with the plasma membrane. Microvesicles (sometimes also referred to as ectosomes or microparticles) are large EVs, ranging between 100-1000 nm in diameter, which are secreted by shedding or budding from the plasma membrane [2, 3]. Recently, some cancer cells have been found to secrete very large EVs (1-10 μm) called large oncosomes. These are due to the shedding of non-apoptotic plasma membrane blebs, which are characteristic of fast-migrating “aboeboid” tumour cells [4]. Currently, there is no consensus about whether these EVs represent a subclass of microvesicles or an entirely new class of EVs. Apoptotic bodies are heterogeneous EVs, which contain cytoplasm with condensed organelles and/or nuclear fragments. They are released into the surrounding extracellular space by apoptotic/dying cells [1]. *In vivo,* apoptotic bodies are quickly cleared by macrophages and other phagocytes [5]. Although they have been found to carry miRNAs, which can be functionally active in the recipient cells [6], they are structurally and functionally very different from live cell-derived EVs and will not be discussed in this review.

Although exosomes, microvesicles and oncosomes have distinct physical and biochemical properties, so far, no markers that can unambiguously distinguish these types of EVs have been identified. Furthermore, the current methods used for the fractionation of EVs cannot reliably separate various types of EVs [3]. Furthermore, recent studies have uncovered substantial differences in the EV biogenesis of various cell types. This suggests that discriminating these types of EVs could be more complex than initially thought [3, 7]. Therefore, in the current review, we will use the term EV to designate all types of live cell-secreted vesicles.

Cancer-derived EVs have been shown to carry a variety of lipids, proteins, mRNAs, non-coding and structural RNAs and even extra-chromosomal DNA [8][Bibr bibr9-60522]–[10]. The molecular content of EVs partially reflects that of the parent cells. However, studies have shown that they are enriched in certain molecules, indicating the existence of specific mechanisms that sort cargo into EVs [11, 12]. Overall, these mechanisms are poorly understood and are likely to be related to the mode of EV biogenesis. The sorting of specific proteins into EVs can be mediated by the endosomal sorting complex for transport (ESCRT) machinery [13] or ESCRT-independent mechanisms such as tetraspanin [14] or ceramide-dependent pathways [15]. The sorting of RNAs into EVs can be mediated by the interaction of specific RNA-binding proteins, such as hnRNPA2B1, with cis-acting elements in the RNA sequence [12, 16].

EVs can be taken up by recipient cells and trigger diverse biological effects. Therefore, they have emerged as important mediators of intercellular communication, both in normal physiological processes and in the development of various diseases [8, 9], [17]. In cancer, EVs have been shown to mediate paracrine signalling between cancer cells, crosstalk between tumour and microenvironment, contribute to the formation of the pre-metastatic niche and interfere with the anti-tumour immune response. However, most of the data regarding their role in cancer come from *in vitro* studies and it is not entirely clear which of the uptake mechanisms are recapitulated *in vivo*. Most data are concerned with the fate of EVs when they are internalized by various cell types, as well as what mechanisms govern the trafficking of EVs in the body. In this review, we summarize studies that aim to assess the tissue distribution, trafficking, clearance and uptake of cancer-derived EVs *in vivo*. We also discuss the impact that they have been shown to have, both locally and systemically.

## 2. Tissue Distribution of Cancer-derived EVs

EVs have been found in various biological fluids, including blood, milk, urine, saliva, etc. [18]. Here, they represent a heterogeneous mixture of EVs derived from various cell types. Several lines of evidence suggest that cancer-derived EVs can be released into the circulation or other biofluids of cancer patients. At first, cancer patients have been found to have higher levels of circulating EVs, compared to healthy controls [19][Bibr bibr20-60522]–[21]. Secondly, EVs isolated from the biofluids of cancer patients or tumour-bearing animals were shown to contain cancer-associated markers, such as Melan-A [19], TYRP2 [22] and CA19-9 [23], and amplified or mutated oncogenes [9, 24]. However, until recently, very little was known about the half-life, clearance, trafficking and tissue distribution of cancer-derived EVs in the body. Data from *in vivo* studies that address these issues have only started to accumulate over the last few years. The main findings of these studies are summarized in Table 1.

**Table 1.** Studies investigating EV biodistribution and functions *in vivo*

Cell Line	Animal Line	EV Labelling	Injection Site	Detection Method	Results	Ref.

Exogenously Administered EVs into Cancer-free Animals						
TS/A murine mammary tumour	BALB/c	PKH67	i.v.	Flow cytometry	EVs are taken up by bone marrow CD11b ^+^Gr-1^+^ cells; suppress myeloid cell differentiation into DCs.	[26]

EL-4 mouse lymphoma	C57BL/6	IRDye800	i.p.	LI-COR imager	EVs detected in the liver, lung, kidney and spleen, taken up by CD11b^+^Gr-1^+^ cells.	[28]

B16-BL6 mouse melanoma	C57BL/6	gLuc-lactadherin	i.v. via tail vein	gLuc activity measurement	Half-life of EVs in the blood is ~2 minutes At 10 to 60 minutes after injection, EVs are distributed mainly to the liver and lungs; at 4 hours – lungs and spleen.	[30]
			
	BALB/c	gLuc- lactadherin	i.v. via tail vein	LAS3000 IVIS		
			
	C57BL/6	PKH26	i.v. via tail vein	Fluorescent microscopy		

HEK293T human embryonic kidney	Athymic nude mice	gLuc	i.v. via retro-orbital vein	Bioluminescence imaging	30 minutes after injection, EVs are distributed to the spleen, liver, lungs and kidneys; actively taken up by liver and lung cells but not spleen cells. EVs are eliminated via hepatic and renal routes.	[27]
				
		Biotin-Alexa680-streptavidin	i.v. via tail vein	FMT imaging		

B16-F10 mouse melanoma	C57BL/6	SPION5	Footpad	*In vivo* MRI	EVs home to the subcapsular sinus of lymph nodes.	[33]

MDA-MB-231 human breast cancer	Nude mice	DiI	i.v. via tail vein	Flow cytometry, IF	EVs are internalized by macrophages in the lungs and brain, resulting in the activation of NF-κB pathway.	[32]

K562 human chronic myelogenous leukaemia	SD rats	n.a.	i.v. via tail vein	n.a.	EVs deliver hybrid BCR/ABL DNA to normal neutrophils; administration of EVs induce CML phenotype in mice and rats.	[36]
					
	NOD/SCID mice		i.v. via tail vein	n.a.		

**Exogenously Administered EVs into Cancer Bearing Animals**						

BSp73ASML BDX rat pancreatic adenocarcinoma	BDX rats	n.a.	Footpad – first EVs, then cells	n.a.	EVs from metastatic cells support the metastatic spread of non-metastatic cells to lymph nodes and lungs.	[37]

B16-F10 mouse melanoma	C57BL/6 albino; i.v. injection of melanoma cells	DiR	Footpad	IVIS	EVs home to sentinel lymph nodes and enhance migration of melanoma cells to EV-rich sites in lymph nodes.	[29]

B16-F10 and B16-F1 mouse melanoma	C57BL/6 mice with orthotopic B16-F10 tumours	PKH67	i.v.	Confocal microscopy	EVs home to the lungs, bone marrow, liver and spleen. EVs enhance metastasis by bone marrow education via the transfer of MET to the bone marrow progenitors.	[22]

HEK293T human embryonic kidney	RAG2^−/−^ mice with breast cancer (HCC70) xenografts	DiR	i.v. via tail vein	IVIS	EGFR-targeted EVs home to tumour microenvironment and can deliver miRNAs to EGFR-expressing breast cancer cells.	[31]

HEK293T human embryonic kidney	Athymic nude mice with glioma xenograft	gLuc	i.v. via tail vein	Bioluminescence imaging	Similar amounts of EVs are found in tumours, spleen and liver.	[27]

4T1 mouse mammary tumour	BALB/c with 4T1 orthotopic tumour	DiR	i.v., i.t.	IVIS200	EVs are taken up in the liver and spleen, very little amounts travel to the tumour. Slower uptake and clearance in mice with impaired innate immunity. Intratumourally administered EVs stay associated with tumour.	[38]
						
	Nude mice with 4T1 orthotopic tumour					
						
	NOD.CB17-Prkdc^scid^/J with 4T1 orthotopic tumour					

**Endogenously Produced EVs from Genetically Engineered Cancer Cell Lines**						

MMT-060562 mouse breast cancer; MDA-MB-231 human breast cancer	Nude mice with orthotopic MMT tumours or MDA-MB-231 xenografts	CD63-GFP	n.a.	CLSM imaging	Breast cancer cells secrete EVs in the primary and metastatic tumour microenvironment and blood circulation; EVs are taken up by cancer cells and CAFs.	[34]

MDA-MB-231 human breast cancer	Nude mice with MDA-MB-231 xenograft	Human CD63	n.a.	IHC	EVs are taken up by macrophages in the lung, brain and lymph nodes; induce inflammatory processes in tumour microenvironment and axillary lymph nodes.*	[32]

H460 human lung cancer	Nude mice with H460 xenograft	hCD63-GFP	n.a.	Immunomagnetic separation, RT-PCR	Human cancer-derived EVs carry mRNAs and are detectable in the blood and saliva.	[35]

gLuc – *Gaussia luciferase*; FMT – fluorescence mediated tomography; i.v. – intravenous; i.p. – intraperitoneal; EV – extracellular vesicle; DC – dendritic cell; BM – bone marrow; MDMC – monocyte derived myeloid cell; IVIS – *In Vivo* imaging system; SPION5 – super paramagnetic iron oxide nanoparticles; MRI – magnetic resonance imaging; PLN – Poplietal lymph node; IF – immunofluorescence; CLSM – confocal laser scanning microscopy; IHC – immunohistochemistry.						

In these studies, two different approaches for studying the biodistribution of EVs have been exploited. One is based on administering exogenous EVs into the circulation of experimental animals, while the other is based on tumour models that produce labelled EVs endogenously. Most of the studies that used exogenously produced EVs isolated them from a cell culture medium by differential ultracentrifugation and sucrose gradient, as described by Thery et al. 2006 [25]. After the intravenous administration has been conducted, the EVs are tracked *in vivo*. This is carried out either by labelling the EVs with fluorescent membrane dyes, such as DiI, PKH67 and PKH26 [22, 26][Bibr bibr27-60522][Bibr bibr28-60522][Bibr bibr29-60522][Bibr bibr30-60522][Bibr bibr31-60522]–[32], and loading them with superparamagnetic iron nanoparticles (SPION5), allowing magnetic resonance tracking [33]; or by using EVs engineered to display a membrane reporter [27, 30], [34].

EV reporter systems have been created by the genetic engineering of cell lines that produce EVs with membrane-anchored Gaussia luciferase (gLuc), biotin acceptor peptide (BAP) [27] or green fluorescent protein (GFP) [34]. Studies that are based on endogenously produced EVs have used either genetically engineered cancer cell lines, which produced GFP-tagged CD63 [34, 35], or human cancer xenografts in mice, where cancer-derived EVs were located by detecting human CD63 [32]. The EV detection methods vary depending on the method of EV labelling and the specific aim of each study. *In vivo* imaging system (IVIS) is the method of choice for the analysis of EV tissue distribution. However, studies that focus on the specific effects caused by cancer-derived EVs in specific organs also used flow cytometry, microscopy, immunohistochemistry, gLuc activity measurements, magnetic resonance, etc.

In cancer-free animals, exogenously administered cancer-derived EVs were distributed mainly to the liver, lungs, kidneys and spleen [28, 30] and were also detected in the lymph nodes [29, 33] and bone marrow [22, 26]. EVs derived from human embryonic kidney cells (HEK293T) were predominantly localized in the spleen, followed by the liver, lungs and kidneys. Additionally, lower amounts of EVs were also detected in the brain, heart and muscle [27]. However, when the EV-injected animals were transcardially perfused with PBS before collecting the organs, the highest amount of EVs was detected in the kidneys and not the spleen, followed by the liver and lungs. This suggests that EVs are actively taken up by the kidney, liver and lung cells but not the spleen cells. It also indicates that the accumulation of EVs in the nonperfused spleen may be due to the uptake of EVs by circulating lymphocytes and macrophages [27]. In the liver and lungs, they are likely to be taken up and degraded by phagocytic cells such as Kuppfer cells and alveolar macrophages [27]. However, a portion of the EVs may also be internalized by the kidney cells and released into the urine [27]. A recent study by Cai et al. (2014) demonstrated that, when injected in rats eliciting some characteristics of CML, EVs derived from the CML cell line K562 transferred BCR-ABL hybrid gene to normal neutrophils. This suggests that EV-mediated transfer of oncogenes may represent a novel mechanism of tumourigenesis [36].

Considering that cancer patients have substantially higher levels of EVs in the blood than healthy individuals [19][Bibr bibr20-60522]–[21] and that cell-free RNAs, part of which are likely to be packaged into EVs, are remarkably stable in the patients' blood [39], it seemed plausible that EVs should be very stable in the biofluids. Unexpectedly, when injected in the blood circulation of immunocompetent mice, murine melanoma-derived EVs had a half-life of only approximately two minutes. Furthermore, they were cleared from the circulation within four hours [30]. Similarly, when injected into athymic nude mice [27], HEK293T-derived EVs had a half-life of less than 30 minutes *in vivo* in most tissues. Moreover, lymphoma-derived EVs were taken up by CD11b^+^Gr-1^+^ cells within one hour after the injection into immunocompetent mice [28]. Such a short half-life of exogenously administrated EVs was very surprising and has to be taken into account when designing studies for the identification of EV-associated cancer biomarkers.

In tumour-bearing animals, both the exogenously administered EVs and endogenously produced EVs have also been found to accommodate the tumour microenvironment, lymph nodes and bone marrow. However, in such studies, the largest part of intravenously administered EVs was rapidly cleared from the circulation. Exogenously administered HEK293T-derived EVs accumulated in xenograft tumours at similar levels than the liver and spleen at 60 minutes post-injection [27]. Nevertheless, it remained unclear which cell types bind or internalize the EVs. Several other studies suggest that, in the tumour microenvironment, cancer-derived EVs can be internalized by other cancer cells, as well as by surrounding cells like cancer-associated fibroblasts (CAFs), endothelial cells and other stroma cells [31, 34], [40]. A number of studies have demonstrated that cancer-derived EVs are released into the blood circulation and are trafficked to the lymph nodes, bone marrow and lungs. Here, they promote metastatic niche formation and enhance the metastatic spread of cancer cells to these organs [22, 29], [34, 37]. Furthermore, when taken up by macrophages, cancer-derived EVs were shown to induce an inflammatory response [32]. Taken together, these studies provide a solid basis for the concept that cancer-derived EVs promote cancer development and progression *in vivo* by inducing various biological effects, both locally and systemically.

## 3. Uptake of EVs in Tumour Microenvironment

It has become clear that, within the tumour microenvironment, the cellular composition is complex. Furthermore, relationships between different cell types are no less sophisticated than those in any healthy organs [41]. Therefore, the role of intercellular communication in the acquisition of various cancer phenotypes, invasive growth and metastasis and drug resistance is increasingly recognized. It can be mediated by soluble signalling molecules, cell-cell or cell-matrix adhesion, gap junctions and EVs [42]. In the tumour microenvironment, cancer-derived EVs have been shown to be taken up by other cancer cells and stromal cells, such as fibroblasts, as well as endothelial cells and tumour-infiltrated immune cells. EVs can exert their effects in the recipient cells either by binding to the cell surface receptors or delivering their content inside the recipient cell. Contrary to the single-molecule signals, EVs have the potential to affect multiple signalling pathways inside the recipient cell. Hence, they provide more efficient means for phenotypic reprogramming or synchronizing the physiological state of the surrounding cells [43, 44]. Internalization can occur either through a fusion with the cell membrane [45], endocytosis and micropinocytosis [46, 47] or phagocytosis [48]. However, it is not yet entirely clear which of these mechanisms lead to the degradation of EV components and which, eventually, result in the release of EV content into the cytoplasm. Here, it can alter the physiological functions of the recipient cell. The recipient cell specificity is likely to be determined by the composition of adhesion molecules and lipid content on the surface of EVs and the respective ligands on the cellular surface [49][Bibr bibr50-60522][Bibr bibr51-60522][Bibr bibr52-60522]–[53]. For example, the internalization of glioblastoma-derived EVs was found to depend on the expression of heparan sulfate proteoglycans on the recipient cells [50]. Meanwhile, the uptake of rat tumour-derived EVs by specific cell types depended on the expression of tetraspanin Tspan8 and integrin α4 on the EVs [54]. Interestingly, low extracellular pH (pH 6.0) has also been shown to increase the release and uptake of melanoma-derived EVs [45]. This finding is particularly significant for understanding the EV function in the tumour microenvironment, as hypoxia and extracellular acidosis are common features of the vast majority of solid cancers. Extracellular acidosis arises by switching metabolism to glycolysis, resulting in the increased production and excretion of acidic metabolites, such as lactic and carbonic acids, and can be as low as 5.9 [55]. Recently, low pH-dependent EV-mediated elimination of cisplatin was also shown to serve as a mechanism of chemoresistance to cisplatin [56].

## 4. Paracrine Effects Caused by Cancer-derived EVs in the Tumour Microenvironment

### 4.1 Cancer Cell Cross-talk

**Figure 1.**
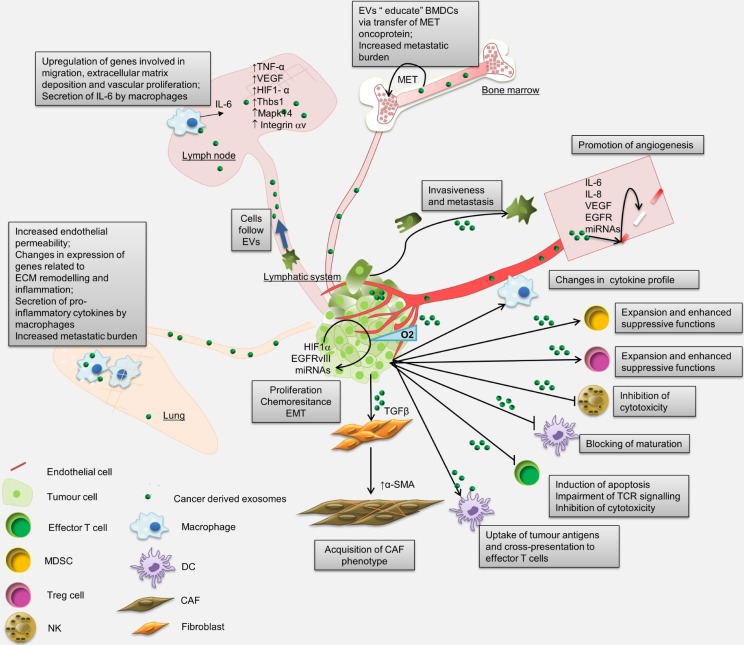
Local and systemic effects that are triggered by cancer-derived EVs. Locally cancer-derived EVs have been reported to promote proliferation, invasiveness and chemoresistance, and to induce EMT in cancer cells in a paracrine manner, and to stimulate angiogenesis and reprogramming of stromal cells into CAFs. Systemically, cancer-derived EVs have been shown to contribute to the generation of metastatic microenvironment by reprogramming BMDCs, regulating gene expression in the lungs and lymph nodes and modulating anti-tumour immune response. BMDCs, bone marrow-derived cells; ECM, extracellular matrix; EMT, epithelial-mesenchymal transition; CAF, cancer-associated fibroblast; DC, dendritic cells; MDSC, myeloid-derived suppressor cell; NK, natural killer cell; TCR, T cell receptor.

The uptake of cancer-derived EVs by other cancer cells can lead to increased invasiveness and metastatic potential [57][Bibr bibr58-60522][Bibr bibr59-60522]–[60], anchorage-independent growth [24, 61], proliferation and chemoresistance [60, 62], [63], as well as inducing the epithelial-mesenchymal transition (EMT) [59, 64] (Figure 1). Moreover, cancer-derived EVs are shown to drive the oncogenic conversion of non-tumourigenic cells [65, 66]. These effects can be mediated by the transfer of functionally active proteins, such as HIF1α [59], EGFRvIII [67], miRNAs [61, 63] and possibly, other non-coding RNAs, mRNAs and fragments of genomic DNA carrying various cancer genes and transposable elements [9, 68], [69].

In 1980, Poste and Nicolson provided the first experimental evidence that EVs, shed from highly metastatic melanoma cells, could increase the metastatic potential of poorly metastatic melanoma cells [57]. Later on, several independent studies confirmed these findings *in vitro* and *in vivo*. A study by Hao et al. (2006) demonstrated that, when i.v. was injected into C57BL/6 mice, the EVs released from highly metastatic melanoma cell line were taken up by poorly metastatic cells, which acquired the capacity to form metastatic colonies in the lungs. Similarly, EVs derived from highly invasive (but not from non-invasive) triple-negative breast cancer cells significantly increased the proliferation, migration and invasion capacity of other breast cancer cell lines [60]. In line with this, EVs from prostate cancer (PC) patients' sera have been found to enhance the proliferation and invasion of PC cell lines [62]. A study by Aga et al. (2014) demonstrated that EVs derived from EBV-infected nasopharyngeal carcinoma contained the functionally active transcription factor, HIF1α. Furthermore, the uptake of HIF1α-containing EVs (but not EVs containing mutant HIF1α) resulted in the downregulation of E-cadherin and upregulation of N-cadherin in recipient cells [59]. Changes in E- and N-cadherin expression are markers for EMT that confer mesenchymal properties to epithelial cells. This is associated with invasion and metastasis, as well as the acquisition of cancer stem cell phenotype [70]. Furthermore, EVs from drug-resistant cell line variants have been shown to confer resistance to non-resistant PC or breast cancer cells [62, 63].

Al-Nedawi et al. (2008) demonstrated that glioma cells with a mutated form of epidermal growth factor receptor (EGFRvIII) release it via EVs, which were taken up by indolent glioma cells [24]. This resulted in the activation of MAPK and Akt signalling pathways. It also led to changes in the expression of EGFRvIII-regulated genes, resulting in the morphological transformation and increase in anchorage-independent growth capacity [24]. Furthermore, breast and colorectal cancer (CRC) cells have been shown to release Amphiregulin (AREG), an EGFR ligand, via EVs [71]. When taken up by breast cancer cells, EV-packaged AREG displayed greater membrane stability and increased invasiveness. Interestingly, the AREG level in EVs correlated with the mutant KRAS status of the donor cells [71]. Later on, the same group demonstrated that mutant KRAS status affects the composition of the EV proteome. When taken up by CRC cells with wt KRAS, EVs derived from CRC cells with mutated KRAS contained many tumour-promoting proteins, including KRAS and EGFR, and enhanced colony formation [72]. Another study demonstrated that EVs derived from a hepatocellular carcinoma (HCC) were enriched in specific miRNAs that could be taken up by other HCC cells. This resulted in the modulation of TAK1 signalling pathway and enhanced anchorage-independent growth [61].

A recent study by Melo et al. (2014) highlights a novel mechanism that could potentially impact our understanding of the physiological role of cancer-derived EVs. This study demonstrated that, in contrast to those produced by normal cells, breast cancer EVs bear a RISC-loading complex. This is associated with pre-miRNAs that are capable of cell-independent miRNA biogenesis. These EVs were able to mediate the rapid and efficient silencing of target mRNAs of recipient cells and, importantly, were shown to have a protumourigenic effect on non-tumourigenic epithelial cells. It was demonstrated that the impact was dependent on the presence of Dicer within cancer EVs [65]. The authors proposed that cancer EVs are capable of inducing an oncogenic “field effect” by subjugating neighbouring normal cells to cooperate in cancer progression. In addition, another excellent study by Abd Elmageed et al. (2014) showed, for the first time, that EVs produced by prostate cancer cells are capable of inducing a neoplastic transformation of tumour-trophic mesenchymal stem cells. The EV-primed adipose-derived tissue stem cells underwent a mesenchymal-to-epithelial transition, adopted genetic instability and oncogenic transformation, and were able to form tumours *in vivo*. This was achieved through the oncogenic factor transfer by prostate cancer cell-derived EVs, which included oncogenic miRNAs, K-*ras* and H-*ras* transcripts and oncoproteins [66].

However, several recent findings have challenged the paradigm of the pro-tumourigenic role of cancer-derived EVs, suggesting that cancer-derived EVs can also have opposite or, so far, unknown roles. For example, a recent study by Gabriel et al. (2013) demonstrated that EVs derived from various cancer cell lines and PC patients' plasma contained a functionally active tumour-suppressor protein PTEN that suppressed the proliferation of PTEN-deficient recipient cells [107]. As the authors did not detect it in the EVs derived from normal cells and plasma from healthy individuals, they suggested that this might represent a mechanism used by cancer cells to downregulate PTEN level [107]. Meanwhile, another study found a functional PTEN protein in EVs, produced by mouse embryonic fibroblasts and human embryonic kidney cells [108]. This suggests that this could be a more wide spread phenomenon, provoking questions regarding its biological significance in normal physiological processes and cancer. Moreover, it remains to be determined which cell types actively take up PTEN-containing EVs *in vivo* and whether they exert the same physiological effects in cancerous and normal cells.

Taken together, several lines of evidence strongly support the concept that cancer-derived EVs act paracrinally to synchronize the physiological state in subpopulations of cells. They do this by delivering signalling molecules that are not endogenously expressed in the recipient cells and thus, driving the cancer progression. Furthermore, they may even induce the acquisition of cancer cell phenotype in non-malignant cells. However, various EV subpopulations differ in their molecular content and may cause opposite effects in the recipient cells. Thus, further studies that dissect the heterogeneity of EVs and the recipient cell selectivity are urgently needed.

### 4.2 Promotion of Angiogenesis

Cancer-derived EVs have also been shown to promote angiogenesis. A number of independent studies have demonstrated that cancer-derived EVs can be taken up by the endothelial cells *in vitro* and *in vivo*. This results in morphological changes, migration and proliferation of endothelial cells, tube formation and neovascularization [40, 67], [73][Bibr bibr74-60522][Bibr bibr75-60522][Bibr bibr76-60522][Bibr bibr77-60522]–[78]. Apparently, these effects can be mediated by the transfer of angiogenic proteins [73] and oncogenic proteins [67], as well as various mRNAs and miRNAs [40, 78], [79]. While the angiogenic proteins, such as angiogenin, IL-6, IL-8, TIMP-1 and VEGF, are likely to impact in a paracrine manner, the delivery of oncogenic EGFR to the endothelial cells was shown to trigger the endogenous expression of VEGF, followed by the autocrine activation of VEGF receptor-2 signalling [67]. Several studies emphasize the role of EV-shuttled miRNAs in the endothelial cell migration and neovascularization. A study by Umezu et al. (2012) demonstrated that the uptake of leukaemia cell-derived EVs carrying miR-92a enhanced endothelial cell migration and tube formation [79]. Meanwhile, Zhuang et al. (2012) showed that tumour-secreted miR-9 triggered the activation of JAK-STAT pathway in the endothelial cells, resulting in enhanced migration and tumour angiogenesis [78]. Furthermore, the administration of anti-miR-9 or JAK inhibitors suppressed these effects *in vitro* and *in vivo* [78]. Likewise, the treatment of mice carrying human carcinoma xenografts with Diannexin, a drug that binds phosphatidilserine and blocks EV exchange, resulted in the reduction of tumour growth rate and microvascular density [67].

Collectively, these studies demonstrated, both *in vitro* and *in vivo,* that cancer-derived EVs have a pro-angiogenic capacity and therefore, might represent an attractive target for therapeutic intervention. However, to date, it is not entirely clear at what stages of maturation endothelial cells are targeted by cancer-derived EVs *in vivo*.

### 4.3 Acquisition of CAF Phenotype

Among the different components of the tumour stroma, cancer-associated fibroblasts (CAFs) are one of the main elements. CAFs are defined as all the fibroblastic, non-neoplastic, non-vascular, non-epithelial and non-inflammatory cells with a stable karyotype found in a tumour [80]. CAFs promote tumour progression by secreting soluble growth factors, cytokines and chemokines that stimulate proliferation and migration of cancer cells, induce angiogenesis, modify tumour metabolism, stimulate acquisition of cancer stem cell phenotype and modulate the immune response [80, 81]. CAFs can originate from resident tissue fibroblasts, mesenchymal stem cells, myofibroblasts and even epithelial and endothelial cells via EMT or EndMT, respectively [80]. This process is accompanied by persistent changes in their gene methylation pattern [82, 83]. Once the CAF phenotype is acquired, two autocrine signalling loops, mediated by TGF-β and SDF-1 cytokines, maintain them in this differentiation state in an autocrine manner [84].

A growing body of evidence suggests that cancer-derived EVs play a crucial role in the reprogramming of these cells into CAFs via the transfer of TGFβ. Thus, for instance, TGFβ1 containing PC-derived EVs was found to be taken up by primary lung fibroblasts, resulting in their differentiation into tumour-promoting CAFs [85, 86]. These cells supported angiogenesis *in vitro* and tumour growth *in vivo*. Interestingly, this effect could not be achieved by using soluble TGFβ1 and appeared to depend on heparan sulphate chains on the EV surface. Moreover, EV-deficient (Rab27a knock-down) cancer cells failed to achieve activation of the tumour stroma [86]. Likewise, gastric cancer-derived EVs were shown to trigger the differentiation of umbilical cord-derived mesenchymal stem cells into CAFs by the transfer of TGFβ and the subsequent activation of TGFβ/Smad pathway [87]. Furthermore, breast cancer-derived EVs were found to convert adipose tissue-derived mesenchymal stem cells into CAFs expressing various tumour-promoting factors [88].

Hence, cancer-derived EVs seem to play a crucial role in the induction of CAF phenotype via the transfer of TGFβ.

## 5. Systemic Effects Caused by Cancer-derived EVs

### 5.1 Formation of Pre-metastatic Niche

The first evidence that cancer-derived EVs can contribute to the generation of metastatic microenvironment was provided by Jung et al. (2009) [37]. In this study, rats received injections of a conditioned medium containing EVs and a soluble matrix obtained from highly metastatic pancreatic cancer cells, followed by the injection of the respective cancer cells. This showed that the conditioned medium promoted the settlement of a non-invasive variant of these cells in the lymph nodes and lungs. This suggested that highly metastatic cells deliver messages that elicit alterations in pre-metastatic organs. This allows the homing, settling and growing of poorly metastatic cells [37]. Another study demonstrated that, when injected in the mouse footpad, mouse melanoma-derived EVs, home to sentinel lymph nodes, enhance the migration of melanoma cells to the EV-rich sites in the lymph nodes. In the lymph nodes, EVs were found to regulate the expression of a variety of genes involved in migration, extracellular matrix deposition and vascular proliferation [29]. Moreover, EVs derived from putative renal cancer stem cells were found to increase the number of lung metastases, when intravenously injected in SCID mice [74]. An elegant study by Peinado et al. (2012) demonstrated that, when injected in mice, EVs derived from highly malignant mouse melanoma, home to the lungs and bone marrow, enhance endothelial permeability at pre-metastatic sites and promote the development of distant metastasis. These EVs were found to “educate” bone marrow-derived cells (BMDCs) by transferring the MET oncoprotein, resulting in the activation of the MET pathway in BMDCs. They become conditioned to support tumour vasculogenesis, invasion and metastasis. Furthermore, higher amounts of MET were found in circulating EVs isolated from patients with stage three and four melanoma than in healthy controls. This shows that this finding may also have relevance in a clinical setting [22].

A number of preclinical studies suggest that cancer-derived EVs have a systemic effect on the conditioning of a pre-metastatic niche and hence, set the basis for a new therapeutic strategy. However, it remains to be determined whether or not this type of signalling represents a common mechanism of metastasis in various human cancers.

### 5.2 Modulation of Anti-tumour Immune Response

Cancer-derived EVs have been reported to both stimulate and suppress anti-tumour immune responses [11]. They are shown to contain tumour-associated antigens, such as CEA and MART1, and are efficiently taken up by dendritic cells. In turn, these cross-present the antigens to CD8^+^ T cells, resulting in potent anti-tumour effects [89, 90]. Moreover, as they bear MHC class I molecules, it has been suggested that they could directly stimulate CD8^+^ T cells [11]. In fact, several pre-clinical and clinical studies based on the immunization with cancer-derived EVs, in combination with various cytokines, have shown the induction of beneficial tumour-specific CD8^+^ T cell responses. Such studies suggest that they may represent an attractive approach for cancer immunotherapy [91][Bibr bibr92-60522][Bibr bibr93-60522]–[94].

On the other hand, increasing evidence suggests that cancer-derived EVs can suppress the anti-tumour immune response in a variety of ways. For instance, melanoma-derived EVs have been shown to be enriched for FasL and induced Fas-mediated apoptosis in T cells [95]. Subsequent studies have described similar immune evasion mechanisms, mediated by FasL or TRAIL expression, on EVs in prostate and colorectal cancer and glioma [96][Bibr bibr97-60522][Bibr bibr98-60522]–[99]. Moreover, FasL expression can also lead to the TCR impairment due to the downregulation of CD3-ζ chain, which has been reported in ovarian [100, 101] and head and neck squamous carcinoma patients [98]. Contrary to the effector T cells, CD4^+^CD25^high^Tregs are resistant to FasL-induced apoptosis and cancer-derived EVs have been reported to stimulate the expansion and suppressive functions of Tregs [98, 102], [103]. Ovarian cancer-derived EVs have been shown to promote the proliferation of CD4^+^CD25^+^FOXP3^+^T cells, convert CD4^+^CD25^neg^ T cells into CD4^+^CD25^+^Tregs and upregulate Treg suppressor functions (e.g., production of perforin, Granzyme B, IL-10, etc.), when added to the culture of peripheral blood T cells obtained from healthy donors [102]. At least partially, this effect seems to be mediated by EV-transferred TGFβ1. This is because the pre-treatment of malignant-effusion derived EVs, with neutralizing antibodies against TGFβ1, reduced the expansion and suppressive functions of Tregs [104]. However, another recent study demonstrated that CD4^+^CD25^high^Treg expansion and IL-10 secretion is promoted by cancer cell-secreted miR-214 that targets PTEN in CD4^+^ T cells [105].

Other EV-mediated immunosuppressive mechanisms include T cell inhibition via the production of extracellular adenosine by EV-expressed CD39 and CD73 [106], and the downregulation of the activating receptor NKG2D on NK and CD8^+^ T cells by EV-transferred TGFβ [103, 107]. Cancer-derived EVs have also been shown to suppress the cytotoxic activity of NK cells by expressing MICA, which triggers the downregulation of NKG2D from the cell surface and reduces NK cytotoxicity [108].

In addition, cancer-derived EVs have multiple effects on myeloid precursors, dendritic cells and macrophages. Cancer-derived EVs have been shown to block the differentiation of myeloid precursors into dendritic cells and promote the generation of myeloid-derived suppressor cells via Stat3 activation [26, 109], [110]. In turn, this results in the suppression of effector T cell proliferation, activation and cytolytic functions and the induction of Treg cells [109, 111]. In macrophages, melanoma and breast cancer-derived EVs (but not those from non-cancerous cells) have been reported to activate NF-κB signalling and to alter the cytokine and chemokine profile, favouring the production of pro-inflammatory cytokines. However these changes were complex and not consistent with M1 or M2 polarization [32, 112]. Hence, the role of cancer-derived EVs in the macrophage-mediated tumour-promoting or anti-tumour effects is not entirely clear and, presumably, may vary depending on their content and physiological state of their cell-of-origin.

## 6. Concluding Remarks and Future Directions

Collectively, these studies strongly support the paradigm of the cancer-promoting role of cancer-derived EVs. They suggest that inhibition of the formation or uptake of cancer-derived EVs or their components could be a novel therapeutic avenue. In fact, several studies have demonstrated that blocking the production or uptake of EVs or specific miRNAs carried by EVs reduced tumour growth and angiogenesis. This clearly shows a therapeutic benefit [67, 78], [113].

In addition, natural or genetically engineered EVs can be exploited as tools for delivery of virus-like particles or other gene therapy products, allowing to evade pre-existing neutralizing antibodies against the viral vectors and to increase transduction efficiency [114, 115].

Moreover, cancer-derived EVs seem to have very diverse effects on immune cells, which may lead to the stimulation or suppression of anti-tumour immune responses. Hence, a deeper understanding of mechanisms and how they impact the functions of various immune cell subsets could help to develop novel strategies for shifting the balance towards immunostimulatory tumour microenvironment.

However, it remains unclear whether it is possible to entirely stop cancer progression by inhibiting the formation or uptake of cancer-derived EVs. It seems likely that cancers differ in their ability to produce EVs and in the degree to which they depend on the EV-mediated signalling. However, to the best of our knowledge, the levels of EVs and their effects have not been systematically studied during the course of disease progression and compared among different cancer types. Another layer of complexity is added by the heterogeneity of EV biogenesis and the composition of their molecular cargo. Currently, there is great controversy regarding what types of EVs each cell type produces and which of them carry molecular cargo that are capable of eliciting biologically significant effects. For instance, many studies have reported that exosomes are enriched in miRNAs, suggesting that they function as vehicles for the intercellular transfer of miRNAs [8, 116][Bibr bibr117-60522][Bibr bibr118-60522][Bibr bibr119-60522]–[120]. Nonetheless, a recent study by Chevillet et al. (2014) has challenged this view by demonstrating that, on average, most exosomes harbour less than one molecule of a given miRNA. However, it remains unclear whether rare exosomes in the population carry many copies of a given miRNA or whether a larger fraction of exosomes carries a low concentration of miRNAs [121]. In this regard, a recent study by Thakur et al. (2014) demonstrated that exosomes carry dsDNA representing the whole genomic DNA. However, only a subset (~10%) of exosomes contained DNA [122]. Hence, the characterization of EV subpopulations carrying cancer-derived molecular cargo seems to be of paramount importance for designing studies aimed at the discovery of EV-associated biomarkers and therapeutic targeting of EVs.

## 7. Conflict of Interest

The authors declare no conflicts of interest.
